# Medical News and Miscellany

**Published:** 1898-04

**Authors:** 


### Medical News and Miscellany.

**Dr. A. B. Cox** has removed from Bonham to Ladonia, his old home.

---

**Dr. T. J. Dodson** has removed from Bartlett to Sonora, Texas.

---

**Dr. J. B. King** has changed his location from Rancho to Junction, Texas.

---

**Dr. J. C. Jones,** of Gonzales, has been appointed Surgeon General of the United Confederate Veterans of Texas.

---

**For Sale,** very cheap, a Nedofik operating sofa, the same as used by Dr. Wyeth and in the Polyclinic. Address this office.

---

**Dr. W. O. Rodgers,** Sovereign Physician of the Woodmen of the World, died at his home in Omaha, Nebraska, on March 10th.

---

**The** editorial and publication offices of the *North Carolina Medical Journal* have been removed from Wilmington to Winston, N. C.

---

**Alice Mitchell,** the sexual pervert and murderer of Miss Freda Ward, died in the State Insane Asylum at Bolivar, Tenn., on March 31st.

---

**Dr. R. H. Chittenden,** of Yale, has accepted the Chair of Physiological Chemistry in the College of Physicians and Surgeons, New York.

---

**Good practice** and drug store for sale cheap. Good reasons for selling out. Address, P. R., care TEXAS MEDICAL JOURNAL, Austin, Texas.

---

**Dr. Edward B. Jackson,** of Houston, Texas, desires a young physician partner. Must be sober and energetic. Write or call for particulars.

---

**Dr. W. N. Rogers** has removed from Belton to Waco, where he has established a private sanitarium for the treatment of opium, morphine and other habitues.

---

**Dr. D. C. Montgomery,** one of the oldest physicians of the Mississippi delta, and ex-surgeon of Armstrong's Brigade, C. S. A., died at his home in Greenville, Miss., on March 26th.

---

**Dr. J. R. Jordan,** Secretary of the Alabama Medical Association, died at his home in Montgomery, Ala., on March 27th. Dr. Jordan was born in Lexington, Va., on March 4th, 1860.

---

**Brazos Valley Medical Association** will meet in its fifth semi-annual session, at Calvert, Texas, on the second Tuesday in May, and hold two days. The programme will appear in our next issue.

---

**Dr. William L. Nichol,** so long identified with the schools of Nashville, Tenn., as a teacher of medicine, will in future lecture and teach exclusively in the University of the South, at Sewanee, Tenn.

---

**Important Notice.**—One fare round-trip tickets to the meeting of the Texas State Medical Association will be on sale one day only, the 25th of April, 1898. Those who expect to attend should buy their tickets on that date.

---

**Drs. G. W. Emory and Fred B. Johnston,** of Anderson, Texas, have formed a partnership. Dr. Johnston recently located in Anderson, formerly living in East Tennessee. Dr. Emory is at present in New York attending the Polyclinic.

---

**Doctor** (just arrived at the scene of the accident): What on earth are you holding his nose for?

Pat (kneeling beside the victim): So his breath won't leave his body, of course.—*Journal of Practical Medicine.*

---

**The** *London Lancet* states that the University of Aberdeen, Scotland, proposes to confer the honorary degree of LL. D. on Dr. William Osler, M. D., F. R. C. P. Lond., Professor of Medicine in the Johns Hopkins University, Baltimore, U. S. A.

---

**Dr. E. P. Becton,** the popular superintendent of the State school for the blind at Austin, has been elected to deliver the address before the Alumni Association of the Medical Department of the University of Texas, at their annual meeting in Galveston, May next.

---

**The Chair of Diseases of the Eye, Ear and Throat** at the Medical College of Virginia, made vacant by the death of Prof. Charles M. Shields, will be filled at the annual meeting of the Board of Visitors of the College April 21st. All applications, accompanied by credentials, should be forwarded to Christopher Tompkins, M. D., Dean, Richmond, Va.

---

**Torticollis—Treatment.**—This distressing condition may often be alleviated by the administration of conium in large doses—sixty drops of the fluid extract in the course of twenty-four hours—and hypodermics of atropine. The galvanic current applied to the side of the neck oftentimes gives relief, particularly if there is much pain. Massage is also of service.—J. Collins (*Medical Record*, February 12, 1898).—*Monthly Cyclopedia of Practical Medicine.*

---

### Filter the Water.

London has a death rate a fraction of that from which we suffer. Why? Because she has common sense enough to filter the water, originally filthier than ours, which she uses. While our bosses and politicians are "feathering their own nests," annexing Hawaii, and kicking up a fuss with foreign countries; they have not the time to attend to the health of the people whom they misrepresent, and the murdered people meekly die, and the friends of the dead continue to vote for the bosses and their henchmen.—*North American Journal of Diagnosis and Practice.*

---

### Fibroid Tumors and Pregnancy.

At a recent meeting of the Obstetrical Society of France Keiffer read an extended paper upon this subject, in which he drew the following conclusions: Hysterectomy in fibroid uteri in pregnant patients is demanded for four indications: (1) when, independently of pregnancy, the fibroid tumor would make hysterectomy justifiable; (2) when the fibroid occupies such a position that labor would be impossible; (3) when the tumor is degenerating, or suppurating, and when a retained placenta complicates the case; (4) hysterectomy should be performed in a case of labor complicated by fibroid tumor of the uterus after the child has been extracted by Cæsarian section.—*Pacific Medical Journal.*

**Municipal Responsibility for Typhoid Fever.**

According to the *Cleveland Medical Journal* for January, 1898, municipalities will ere long have to be far more careful of the quality of water and other articles furnished for the public use, if the Supreme Court of Wisconsin does not reverse the decision of the lower court. On December 3 a jury in Circuit Court at Stevens' Point, Wisconsin, brought in a verdict for $5000 damages against the Ashland Water Company in favor of the Widow of Lars Green, who died of typhoid fever alleged to be due to contaminated water supplied by the defendant company, which is alleged to have been criminally negligent in providing no purer water. If this principle becomes well established it will have a great effect for good upon all municipalities and all companies engaged in supplying public necessities. —*Medicine.*

